# Performance of the *digene *LQ, RH and PS HPVs genotyping systems on clinical samples and comparison with HC2 and PCR-based Linear Array

**DOI:** 10.1186/1750-9378-6-23

**Published:** 2011-11-18

**Authors:** Jose M Godínez, Sara Tous, Nuria Baixeras, Judith Moreno-Crespi, María Alejo, Marylène Lejeune, Ignacio G Bravo, F Xavier Bosch, Silvia de Sanjosé

**Affiliations:** 1 Unit of Infections and Cancer (UNIC), Cancer Epidemiology Research Program (CERP), IDIBELL. Catalan Institute of Oncology (ICO), (Gran Via 199-203), l'Hospitalet de Llobregat (08907, Barcelona), Spain; 2 Pathology Department, Hospital Universitari de Bellvitge-IDIBELL (Feixa Llarga s/n), l'Hospitalet de Llobregat (08907, Barcelona), Spain; 3 Pathology Department. Doctor Josep Trueta Universitary Hospital - ICO, (Avda. França s/n), Girona (17007), Spain; 4 Pathology Department. Hospital General de l'Hospitalet (Av. De Josep, Molins s/n), l'Hospitalet de Llobregat (08906, Barcelona), Spain; 5 Molecular Biology and Research Section, Hospital de Tortosa Verge de la, Cinta, Institut d'Investigació Sanitària Pere Virgili (IISPV), Universitat, Rovira i Virgili (URV) (C/de les Esplanetes, 14) Tortosa (43500) Spain; 6 Consorcio de Investigación Biomédica En Red en Epidemiología y Salud, Pública, (CIBERESP), Spain

**Keywords:** Papillomaviruses, HPV, genotyping, cervical cancer screening

## Abstract

**Background:**

Certain Human Papillomaviruses (HPVs) are the infectious agents involved in cervical cancer development. Detection of HPVs DNA is part of the cervical cancer screening protocols and HPVs genotyping has been proposed for its inclusion in these preventive programs. The aim of this study was to evaluate three novel genotyping tests, namely Qiagen LQ, RH and PS, in clinical samples with and without abnormalities. For this, 305 cervical samples were processed and the results of the evaluated techniques were compared with those obtained in the HPVs diagnostic process in our lab, by using HC2 and Linear Array (LA) technologies.

**Results:**

The concordances and kappa statistics (k) for each technique compared with HC2 were 98.69% (k = 0.94) for LQ, 98.03% (k = 0.91) for RH and 91.80% (k = 0.82) for PS. There was a very good agreement in HPVs type-specific concordance for the most prevalent types HPV16 (kappa range = 0.83-0.90), HPV18 (k.r.= 0.74-0.80) and HPV45 (k.r.= 0.82-0.90).

**Conclusions:**

The three tests showed an overall good concordance for HPVs detection when compared with HR-HC2 system. LQ and RH rendered lower detection rate for multiple infections than LA genotyping. However, our understanding of the clinical significance of multiple HPVs infections is still incomplete and therefore the relevance of the lower ability to detect multiple infections needs to be evaluated.

## Background

Human Papillomaviruses (HPVs) are among the most common viruses identified in sexually active women all around the world, and persistence of the HPVs infection is a necessary step for the development of cervical cancer [1]. Cervical cancer is the second most common cancer in women, with a yearly incidence of 15.8 per 100,000 (crude rate), causing more than 270,000 deaths in 2008 [2]. Other diseases with clinical relevance are also related to HPVs infection, such as genital warts or intraepithelial lesions and cancer of the vulva, penis, vagina and anus [3-5].

More than 150 different PVs infecting humans have been described [6]. Initially, and on the basis of epidemiological studies of prevalence in cervical lesions, HPVs with genital tropism were classified either as Low Risk (LR-HPVs) or High Risk (HR-HPVs) [7]. Oncogenic types such as HPV16, 18, 31 and 45 are responsible for the vast majority of all cervical cancers worldwide [8], and are also present in other genital lesions (penile or vulvar carcinomas). On the other hand, LR-HPVs, such as HPV6 or HPV11, are usually described as non-oncogenic and are associated to benign lesions such as genital warts, condylomas or low grade genital carcinomas [9].

Because of the clinical and economical relevance of the lesions caused by HPVs infections, screening programs for the early detection of cervical cancer have been established [10,11]. These pathology-preventive programs are based on cytology as a first step and have incorporated more recently HPVs detection as a supplementary test. However, since infection by different HPVs implies differential risk for the development of cervical cancer [7,12,8], it has been proposed that HPVs genotyping should be implemented as part of the HPVs testing for cervical cancer screening programs [13]. Several commercial methods for HPVs detection have been developed and tested [14] in an effort for obtaining robust and easy-to-use technology. Among them, the FDA-approved Hybrid Capture 2 test (HC2; Qiagen, Gaithersburg, MD, USA) is the most used methodology for clinical purposes. HC2 technology has been evaluated in several clinical assays, with better results than cytology regarding sensitivity for HPV-related cytological abnormalities detection [15,16]. HC2 is a HR-HPV-specific test for the detection of 13 HPVs: namely HPV16, 18, 31, 33, 35, 39, 45, 51, 52, 56, 58, 59 and 68. This test detects HR-HPV in clinical samples, but the readout refers to the presence/absence of the tested viruses, and therefore cannot distinguish between single and multiple infections and cannot resolve the presence of multiple HPVs in one sample [17].

HC2 has been proposed and used as a reference methodology for clinical HPV detection [18]. However, no technology has been considered as a "gold standard" for HPV genotyping in clinical samples, so all new proposed technologies need to be compared with previous results for assessing its validity and capacity to detect HPVs.

One of the most used genotyping methods, as well in research and as in clinical use, is the Linear Array (LA; Roche Molecular Systems, Branchburg, NJ, USA). This hybridization, strip-based technique allows to identify 37 different HPVs, [19] spanning HR-HPVs, LR-HPVs and other HPVs with still unclear differential association to malignancy. LA has been used in HPVs detection and genotyping in large epidemiological studies [20,21], and has been proposed as a valid methodology for clinical detection of HPVs in paraffin-embedded samples [22,23].

The present study was designed to compare the performance of three new HPVs genotyping methods, namely *digene *HPV Genotyping LQ Test, *digene *HPV Genotyping RH Test and *digene *HPV Genotyping PS Test, with previous data from our HPVs diagnostic lab. Baseline data were obtained by means of HC2 and LA in the context of screening activities.

## Methods

### Clinical specimens and HC2 screening

Since 2006, Catalonia (Spain) has a population-based program for cervical cancer to regulate cervical cytology frequency and to reincorporate women with poor screening history into the normalized monitorization track. HPVs DNA detection using HC2 was introduced for this later group as an adjuvant of cytology. In the context of this preventive program, HC2 is also indicated in the atypical squamous cells of undetermined significance (ASC-US) triage.

Four hundred (400) cervical samples were obtained in a retrospective way from four different centers participating of this cervical cancer screening program: University Hospital of Girona, Hospital Consortium of Vic, Bellvitge University Hospital and Hospital of Tortosa Verge de la Cinta. The criteria for the selection of the samples were established in the working protocol as follow: 90% of HC2+ samples and 10% of HC2- samples. With this criterion, the samples were randomly selected from the tested population in the four participating centers.

Patients included in this program were women attending regular gynecologic visit, aged >40 years old with no cytology in the five previous years (27.2% of the sample size); women with ASC-US diagnosis in cytology (63.6%); women in a follow-up protocol after surgery or previous HC2+ test 9.2%). The cytological diagnoses were classified according to the Bethesda System (Table 1). The mean age of the involved patients was 37.3 years old (range 17-82). The final sample size was n = 305 samples that were finally analyzed with the five techniques, after excluding samples due to technical issues (e.g. not enough material or invalid samples for some of the protocols). All patients' data were anonymized and the samples were identified with a numerical key.

**Table 1:** HPV DNA results by HC2 at entry and concomitant cervical cytology diagnosis.

	*N*	*%*	N*	%*
***HPV -***	**35**	**8.75**	**35**	**11.48**

***NEGATIVE***	35	8.75	35	11.48

				

***HPV+***	**365**	**91.25**	**270**	**88.52**

***ASC-US***	261	65.25	182	59.67

***HSIL***	9	2.25	7	2.30

***LSIL***	9	2.25	9	2.95

***NEGATIVE***	35	8.75	33	10.80

***MISSING***	51	12.75	39	12.80

***TOTAL***	400	100,00	305	100.00

The first two columns show the data of all selected women. The last two columns (marked as *) show the data of samples finally tested with all the techniques.

"ASC-US": Atypical Squamous Cells of Undetermined Significance; "CIN": Cervical Intraepithelial Neoplasia; "HSIL": High Grade Squamous Intraepithelial Lesion; "LSIL": Low Grade Squamous Intraepithelial Lesion.

The samples were collected in *digene *Specimen Transport Medium (STM) and tested for HR-HPVs by HC2. Briefly, the samples were denatured and then incubated with RNA probes for the HPVs types detected by the HC2 kit. The DNA-RNA hybrids are captured with antibodies attached to the wells of the working plate and the reaction is revealed with chemiluminescent signal. This signal is measured in a microplate luminometer, and quantified as Relative Luminescence Units (RLU) for each tested sample. The result of the test is expressed as RLU relative to the cut-off value of the technique, RLU/CO (HR-HPV negative 0<RLU/CO<0.99; HR-HPV positive RLU/CO≥1).

### DNA isolation

The remaining material from the HC2 denaturation protocol was aliquoted in two subsamples. Each half was used for DNA extraction with the purification system proposed by the manufacturers of the different genotyping systems. For LA genotyping, DNA was extracted with the AMPLICOR AmpliLute DNA Extraction Kit (Roche, reference 03750540 190). For LQ and RH genotyping, DNA was extracted with the QIAamp MinElute Virus Spin Kit (Qiagen, reference 57704). In both cases, the extraction was performed manually, according to the manufacturer's instructions.

### Genotyping

#### 1. - Linear Array

The DNA was first amplified and genotyped with the LA technology as part of a follow-up study. This methodology can amplify 37 different HPVs: HPV6, 11, 16, 18, 26, 31, 33, 35, 39, 40, 42, 45, 51, 52, 53, 54, 55, 56, 58, 59, 61, 62, 64, 66, 67, 68, 69, 70, 71, 72, 73, 81, 82, IS39 (HPV82 subtype), 83, 84, and CP6108 (HPV89 subtype) (Table 2) using the degenerate PGMY5+/6+ PCR system and obtaining biotin-labeled amplimers of 450 bp on the L1 gene [24,19]. The PCR reaction also included an additional primer pair targeting the human β-globin gene, giving a 268 bp fragment as internal control. Genotyping was performed in an automated system, Auto-LiPA 48 (Tecan Austria GmbH, distributed by Innogenetics). The amplimers were denatured and transferred to the typing tray containing the genotyping strips coated with HPVs and β-globin probes. The reaction of the amplimer and the probe appears as a colored line in the strip. The result of each sample was compared with the standard guide to evaluate the presence/absence of the different HPVs.

**Table 2:** HPV genotypes detected by the different techniques

*TEST*	*DETECTED GENOTYPES*
***HC2***	HPV16, 18, 31, 33, 35, 39, 45, 51, 52, 56, 58, 59, 68

***LA***	HPV6, 11, 16, 18, 26, 31, 33, 35, 39, 40, 42, 45, 51, 52, 53, 54, 55, 56, 58, 59, 61, 62, 64, 66, 67, 68, 69, 70, 71, 72, 73, 81, 82, 83, 84, IS39, CP6108

***RH***	HPV16, 18, 26, 31, 33, 35, 39, 45, 51, 52, 53, 56, 58, 59, 66, 68, 73, 82

***LQ***	HPV16, 18, 26, 31, 33, 35, 39, 45, 51, 52, 53, 56, 58, 59, 66, 68, 73, 82

***PS***	HPV16, 18, 45

HC2: Hybrid Capture 2 System; LA: Linear Array.

#### 2. - LQ Test

The *digene *LQ genotyping system is a bead-based xMAP technology for the detection of 18 HR-HPVs: HPV16, 18, 26, 31, 33, 35, 39, 45, 51, 52, 53, 56, 58, 59, 66, 68, 73 and 82 [25,26]. Briefly, the probes for HPV DNA detection are linked to coloured micro-beads, one colour per HPV genotype detected. The micro-beads are scanned by a two-laser system, one of the lasers identifying the bead and the other one detecting the presence/absence of the linked amplimer. An internal control for the detection of β-globin amplimers is included in the beads mix. The extracted DNA is amplified with the GP5+/6+ PCR system, generating biotinylated 150 bp amplicons on the L1 gene. The biotinylated amplimers were mixed with the micro-beads under controlled conditions of temperature and shaking. A conjugate for the detection was then added to the mix and the final dilution read in a plate using a LiquiChip 200 Workstation MDD (QIAGEN, Gaithersburg, USA). The results were analyzed and presented with the Software Package QIAplex MDD (QIAGEN, Gaithersburg, USA).

#### 3. - RH Test

The RH Test is a system for the detection of 18 HR-HPVs: HPV16, 18, 26, 31, 33, 35, 39, 45, 51, 52, 53, 56, 58, 59, 66, 68, 73 and 82 [27,28]. The specific probes are immobilized onto nitrocellulose strips, including one probe for the detection of an internal control using human β-globin amplicon. After amplification with GP5+/6+ PCR, the products are added to the plate containing the strips. Under stringent conditions, the amplicons hybridize with the probes and the result visualized by adding a conjugate and a substrate, whose reaction with the hybrids produces a colored band. The genotyping was performed in an automated system, Auto-LiPA 48 (Tecan Austria GmbH, distributed by Innogenetics).

#### 4. - PS Test

PS Test is a HC2-based genotyping system for the specific detection of the three most significant HPVs, namely HPV16, 18 and 45, and conceived as a "second-step" after performing HC2 and having obtained HR-HPV positive results. The working protocol is similar to HC2 but each sample is only tested against three different sets of HPV-specific probes. Each of these sets is a mix of 25-long oligonucleotides with targets distributed throughout the full-length of the corresponding viral genomes showing no cross-reactivity with LR-HPVs. Briefly, the samples are denatured and incubated with the probes. RNA/DNA hybrids are linked to specific probes (one genotype per well and sample) and the chemiluminiscent signal of the reaction is read and measured by a luminometer. In the same way as HC2, positive and negative calibrators as well as positive and negative controls for each probe are included in the working plate and provide the reference cut off values for calculation of the RLU/CO final results.

### Statistical Methods

HPVs detection rates were calculated for each technique. For the comparison of the results, only HPVs genotypes shared by the different assays were considered, i.e. when probes for the detection of the genotype in the samples are included in HC2/LA but not in other of the analyzed tests, the sample is consider as HPVs negative for the test. For the analyses of single/multiple infections, only HPVs genotypes shared by the compared techniques were included in the comparisons.

Percent of concordance (and 95% Confidence Intervals (CI)), agreement in positives (CI 95%) and agreement in negatives (CI 95%) for each technique were assessed taking as a reference the HC2 and LA results. Cohen's kappa statistic and McNemar's *p*-value were calculated for the comparison of each test with LA results, established as reference value for this comparative study. Kappa index indicates the strength of the concordance between techniques (based on a Normal distribution) and ranks from 0 to 1.00 (<0.20: Poor; 0.21-0.40: Weak; 0.41-0.60: Moderate; 0.61-0.80: Good; 0.81-1.00: Very Good) [29]. Standard error for kappa is calculated by the method described by Fleiss and coworkers 1969 [30]. McNemar's *p*-value indicates whether the discordant cases between LA and each technique are randomly distributed (*p*-value<0.05).

## Results

### Comparison of HPVs detection and overall concordances: HC2 vs LQ/RH/PS

According to HC2 results, 270 of the 305 samples (88.5%) were positive for HR-HPVs. After LA analysis, none of the samples showed LR-HPV single infection, and all HC2-negative samples tested also negative for LA, LQ, RH and PS. Thus, no cross-reactivity with LR-HPV was detected in the analyses of the samples.

Among all the HPVs DNA-positive samples tested with LA, HR-HPVs single and multiple HPVs infections were detected in 44.8% and 55.2% of the cases, respectively.

For the comparison of HPV positivity and type-specific detection rates, only HPVs genotypes common to the compared assays were considered. With this, HPV positivity for LQ was 86, 9%, for RH was 86, 2% and 29, 8% for PS. LQ and RH techniques rendered lower frequencies than LA for multiple infections, (Table 3).

**Table 3:** HPV positive samples, single and multiple infections per assay among the 305 samples included in the study.

	*HPV+*		*Single infections*		*Multiple infections*		*P^&^*
	
TEST (common genotypes)*	*N*	%**	*N*	*%#*	*N*	*%#*	
***HC2/LA vs***. ***LQ or RH***	267	87.54	152	56.93	115	43.07	

***LQ ***	265	86.89	173	65.28	92	34.72	0.048

***RH ***	263	86.23	194	73.76	69	26.24	<0.01

***HC2/LA vs***. ***PS***	110	36.07	101	91.82	9	8.18	

***PS ***	91	29.84	87	95.60	4	4.40	0.278

"*": Common genotypes for HC2/LA vs LQ or RH: HPV16, 18, 26, 31, 33, 35, 39, 45, 51, 52, 53, 56, 58, 59, 66, 68, 73, 82; Common genotypes for HC2/LA vs PS: HPV16, 18, 45 "**": % among total of tested samples (n = 305); "#": % among positive samples; HC2: Hybrid Capture 2 System; LA: Linear Array;"&": p-value for the comparison of the proportion of multiple infections between LA and LQ, RH and PS.

When the three techniques (LQ, RH and PS) were compared with HC2 for positivity, concordance was high: 98.7% (*kappa (k.)= *0.94) for LQ; 98.0% (*k*. 0.91) for RH; and 91.8% (*k*. 0.81) for PS. The agreement of any of the tested technique with HC2/LA, with regard to positivity, ranged between 80.0% and 98.9%. PS was the one with the lowest sensitivity, mainly due to its low HPV16 detection rate in single infections. These concordance values decreased when analyzing separately by single and multiple infections (Table 4).

**Table 4:** Concordance values between LQ, RH and PS techniques for HPV DNA detection using HC2/LA as reference values

	*ASSAY*		
***NEGATIVE vs***. ***POSITIVE***	***LQ (N = 265+/40-)***	***RH (N = 263+/42-)***	***PS (N = 91/214-)***

***% concordance (95% CI)***	98.69 (96.68-99.64)	98.03 (95.77-99.27)	91.80 (88.14-94.62)

***Kappa index (95% CI)***	0.94 (0.88-0.99)	0.91 (0.84-0.98)	0.81 (0.75-0.88)

***Agreement among positives (95% CI)***	98.88 (96.75-99.77)	98.13 (95.68-99.39)	80.00 (71.30-87.02)

***Agreement among negatives (95% CI)***	97.37 (86.19-99.93)	97.37 (86.19-99.93)	98.46 (95.57-99.68)

***NEGATIVE vs***. ***POSITIVE SINGLE vs***. ***POSITIVE MULTIPLE***	***LQ***	***RH***	***PS***

***% concordance***	75.74 (70.53-80.44)	75.08 (69.83-79.84)	89.84 (85.88-92.99)

***Kappa index (95% CI)***	0.59 (0.50-0.67)	0.57 (0.48-0.66)	0.78 (0.71-0.85)

***Agreement among positives single infection (95% CI)***	82.89 (75.95-88.51)	89.47 (83.47-93.86)	78.22 (68.90-85.82)

***Agreement among positives multiple infection (95% CI)***	59.13 (49.57-68.21)	48.70 (39.27-58.19)	33.33 (7.48-70.07)

"CI": Confidence Interval; "% Concordance" and "Kappa index (95% CI)": referred to HC2 result.

In order to evaluate a more clinical approach to the analyses, these were performed including only genotypes detectable by HC2. With this, five possible HR-HPV genotypes were excluded (HPV26, 53, 66, 73 and 82). The only difference with respect to the full analysis was found in multiple infections detection with LQ test. Contrary to the observation with the 18 genotypes analysis, no statistically significant difference was found when comparing LA detection of multiple infection with LQ assay (P value with 18 genotypes = 0.048 vs. P value with 13 genotypes = 0.329).

### Comparison of HPV genotyping results: LA vs LQ/RH/PS

The previous results obtained with LA showed that positives for each of the 37 detectable genotypes, except HPV69 and HPV64, were found in at least one of the samples. The viruses most frequently found were HPV16, 31, 58, 51 and 56 were with the three genotyping methods (Table 5)

**Table 5:** Concordance values by techniques and HPV type

*GENOTYPE*	***LA vs***. ***LQ/RH***	*LQ*			*RH*			***LA vs***. ***PS***	*PS*		
	
	*(n)*	*(n)*	*kappa (95% CI)*	*McNemar's* *p-value*	*(n)*	*kappa (95% CI)*	*McNemar's* *p-value*	*(n)*	*(n)*	*kappa (95% CI)*	*McNemar's* *p-value*
***HPV 16***	90	93	0.898 (0.845 - 0.952)	0.581	91	0.898 (0.844 - 0.952)	1.000	90	73	0.825 (0.753 - 0.896)	0.000

***HPV 16 SI***	36	51	0.720 (0.608 - 0.832)	0.001	60	0.658 (0.543 - 0.772)	0.000	81	69	0.806 (0.728 - 0.883)	0.017

***HPV 16 MI ***	54	42	0.581 (0.455 - 0.707)	0.058	31	0.473 (0.335 - 0.611)	0.000	9	4	0.452 (0.114 - 0.790)	0.125

***HPV 18***	20	25	0.736 (0.588 - 0.885)	0.227	22	0.795 (0.658 - 0.933)	0.727	20	14	0.751 (0.586 - 0.916)	0.070

***HPV 18 SI***	5	8	0.607 (0.292 - 0.923)	0.375	9	0.562 (0.249 - 0.876)	0.219	13	12	0.708 (0.502 - 0.914)	1.000

***HPV 18 MI***	15	17	0.538 (0.324 - 0.752)	0.791	13	0.626 (0.412 - .840)	0.754	7	2	0.439 (0.035 - 0.842)	0.063

***HPV 45***	9	11	0.897 (0.754 - 1.000)	0.500	9	0.886 (0.728 - 1.000)	1.000	9	8	0.818 (0.617 - 1.000)	1.000

***HPV45 SI***	5	6	0.907 (0.727 - 1.000)	1.000	5	1.00 (1.000 - 1.000)	1.000	7	6	0.921 (0.768 - 1.000)	1.000

***HPV 45 MI***	4	5	0.662 (0.302 - 1.000)	1.000	4	0.747 (0.408 - 1.000)	1.000	2	2	0.497 (0.000 - 1.000)	1.000

***HPV 31***	41	39	0.885 (0.806 - 0.963)	0.727	36	0.837 (0.742 - 0.931)	0.227				

***HPV 58***	36	26	0.821 (0.714 - 0.928)	0.020	23	0.757 (0.632 - 0.882)	0.000				

***HPV 51***	34	28	0.785 (0.667 - 0.902)	0.146	21	0.702 (0.561 - 0.843)	0.001				

***HPV 52***	33	21	0.474 (0.304 - 0.645)	0.029	21	0.515 (0.347 - 0.683)	0.023				

***HPV 66***	28	27	0.820 (0.706 - 0.935)	1.000	23	0.808 (0.686 - 0.930)	0.180				

***HPV 53***	28	11	0.486 (0.293 - 0.679)	0.000	8	0.421 (0.221 - 0.621	0.000				

***HPV 39***	25	17	0.796 (0.659 - 0.933)	0.008	17	0.745 (0.594 - 0.896)	0.021				

***HPV 33***	21	22	0.825 (0.698 - 0.952)	1.000	20	0.869 (0.756 - 0.982)	1.000				

***HPV 56***	21	29	0.739 (0.599 - 0.880)	0.039	28	0.756 (0.618 - 0.894)	0.065				

***HPV 59***	20	10	0.651 (0.452 - 0.851)	0.002	10	0.651 (0.452 - 0.851)	0.002				

***HPV 35***	12	11	0.955 (0.866 - 1.000)	1.000	9	0.852 (0.688 - 1.000)	0.250				

***HPV 68***	10	9	0.620 (0.360 - 0.879)	1.000	10	0.586 (0.326 - 0.847)	1.000				

***HPV 73***	9	4	0.608 (0.295 - 0.922)	0.063	2	0.357 (0.000 - 0.717)	0.016				

***HPV 82***	4	6	0.594 (0.231 - 0.956)	0.625	6	0.797 (0.522 - 1.000)	0.500				

***HPV 26***	2	2	0.497 (0.000 - 1.000)	1.000	1	0.665 (0.048 - 1.000)	1.000				

"n": Number of cases HPV type-specific positive; "CI": Confidence interval.

When comparing LA and LQ/RH, 169 samples (55.4%) were totally concordant and only thirteen (4%) were totally discordant. For the rest of the samples partial concordance was detected: i.e. LA showed at least one genotype not detected by LQ/RH. On the other hand, LQ and RH tests detected a higher number of infections than LA for some genotypes, such as HPV16, 18, 45, 56 and 82 for LQ; and HPV16, 18, 56 and 82 for RH (Table 5). For the PS test, which includes only three HPVs genotypes, 74 of the 94 genotyped samples were totally concordant with LA results and 17 were totally discordant. Most of the discordant samples (14/17) were positive for three or more different HPVs genotypes by LA. The exact kappa values and ranges for type-specific concordance comparisons are shown in Table 5.

**Figure 1:**
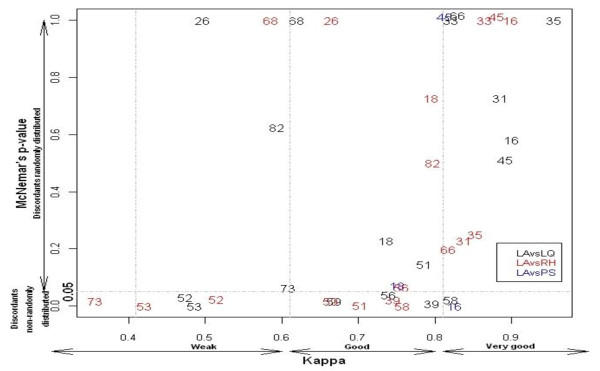
**Type specific concordance between LA and LQ, RH and PS techniques (N = 305)**. The genotyping results for each of the tested techniques vs. LA are presented in a color-coded way. Concordances LA/LQ are presented in black, LA/RH in red and LA/PS in blue.

The main differences were found in the concordance analyses. Although the concordances were good or very good, the distribution of the discordant was non-random for some genotypes, such as HPV16 for PS and HPV51, 56, 58 and 59 for LQ and/or RH. Some other HPVs types showed weak concordance and/or non-randomly distributed discordant, namely HPV26, 52, 53 and 68 and 73 (Figure 1).

## Discussion

In this study we compared the performance of three new HPVs genotyping technologies, namely *digene *LQ, RH and PS Genotyping Tests, with FDA-approved HC2 technique for HPVs positivity/negativity, and with LA methodology for HPVs genotyping, using clinical samples with and without HPVs infection. Statistical analyses of the data showed very good concordance for HPVs detection for all the techniques (range 91.8% - 98.7%), and the kappa indexes of the concordances for the two most relevant HPVs in cervical cancer were between 0.82-0.90 for HPV16 and 0.74-0.79 for HPV18.

HC2 is the most widely used technology for HPVs detection [16,31] and has been proposed as a reference for the comparison of new techniques [18]. It includes probes for thirteen different HR-HPVs. The HC2 protocol performed in our lab cannot detect LR-HPVs, and the results provide exclusively information on the presence/absence of HPVs DNA, without further genotyping ability.

The concordance in HPVs detection is above 90% for all the compared techniques. In the case of agreement in positives LQ and RH tests rendered values of 98.9% and 98.1% respectively while PS test showed an 80.0%, when compared with HC2. Regarding agreement in negatives all LQ, RH and PS displayed values higher than 97%.

The observed differences in the detection rates by assay when compared with HC2 were not statistically significant. Part of the variability could be explained by technical and design issues. First, the study is based on a retrospective design and the working protocols were performed in samples previously obtained. These samples were initially tested for HC2 and LA and aliquots of material, both DNA and denatured STM, were kept frozen at -80°C. For PS test, the material used was the denatured one remaining from the HC2 testing. A decrease in the RLU/CO signal is expected after re-testing the same sample two or more times with HC2 technology [32], this decrease being greater if the material has undergone freeze-thaw cycles. Since both HC2 and PS tests are hybridization-based it may be arguable that they may suffer from the same limitations, which could explain the relative low detection rate of the PS test. No other comparative study with PS test has been published, neither prospective nor retrospective, and the loss in performance should be further studied. For RH and LQ tests, re-extracted DNA was amplified for genotyping. LQ and RH are PCR-based test so that even if the quality and/or the amount of the DNA is not totally preserved, the amplification protocol may still achieve detection rates similar to those with HC2- based tests.

Second, regarding the technical part, two closely-related issues should be considered for the PCR-based tests. On the one hand, DNA quality and purity is a key point in the PCR-based protocols [33] and different isolation procedures may suit best different PCR amplification systems. For PGMY09/11 and GP5+/6+ amplifications, DNA was extracted with silica filter-based commercial kits, ensuring enough purity and quality for downstream applications. On the other hand, two different sets of primers were used and differential assay sensitivity HPV type-specific cannot be excluded. The PGMY9/11 primer set used in LA generates an amplicon of 450 bp, which encompasses the 150 bp amplicon generated with the GP5+/6+ primer set used in LQ and RH tests. Longer amplimers are more sensitive to DNA nicks and fragmentation. This could explain in our case, the higher detection rate of HPV16, 18, 45, 56 and 82 in LQ or RH with respect to LA (3% and 1% higher detection rates for HPV16 using LQ and RH, respectively; 25% and 10% for HPV18). Finally, simultaneous amplification of multiple targets using degenerate primers implies that different HPVs DNA templates compete with each other and with different affinities for the primers. This competition can lead to biases in the results, as has been previously described for HPV16 [27]. In our study, all samples positive for HPV16 and HPV18 with LQ and RH but that had tested negative for both viruses with LA corresponded to multiple infections. We interpret therefore these differences as arising from primers mismatch and from competition between genotypes present in the sample [34].

The proportion of multiple infections detected with LA is higher than with LQ or RH techniques, even after correcting for the different number of genotypes included in the determination (Table 3). Similar findings on the power of LA to identify higher rates of multiple infections as well as to detect a larger number of genotypes in the multiple infections had been previously reported [12]. The clinical relevance of this increased ability to reveal multiple infections still needs to be clarified, as it is also the case for the implications of multiple infections in clinical prognosis. The LQ, RH and PS tests have been developed for clinical settings with the aim of identifying only medically relevant infections [35]. For this purpose, their sensitivity cut-off values lay a higher range than that of LA. As an example, in the case of HPV 58 and HPV59, LA shows lower detection levels for these genotypes than for the rest of detectable genotypes (185 copies/mL and 53 copies/mL respectively). The LQ cut-off value used was the one supplied and recommended by the Luminex software (100 Median Fluorescence Intensity, MFI). Cut-off values as low as 30 MFI have been demonstrated to be useful for HPVs detection, with the trade-off of a concomitant increase in HPVs detection rate [25].

Despite of the good concordances showed in our study, some differences were found in the agreement of the detection rates. Figure 1 shows the graphic representation of the Kappa value-McNemar's p-value relation for each genotype when the techniques are compared. Here we could see how HPV genotypes detected with good concordance between techniques and randomly distributed discordant appeared in the right side of the graphic (e.g. HPV16, 33, 35 and 45). When moving to lower values of Kappa and McNemar, we could find genotypes in the "*Weak concordance - Discordant not random*" region of the graphic (e.g. HPV52, 53 and 73). For HPV52 and 53, technical issues could explain these mismatches. Thus, LA tends to overestimate the detection rate of HPV52 because of the cross-reactivity with other probes in the stripe (e.g. HPV33, 35 and 58). In our case, LA detected 33 HPV52 infections, while LQ and RH systems detected 21 infections each. For HPV53, false negativity has been reported when using the GP primer set [36], as in our case (28 LA vs. 11 LQ vs. 8 RH)

In technical terms, the tests are comparable according to their requirements in sample and in working time. The required sample amount for PCR-based protocols is the same, 10 μL DNA. Furthermore, in the case of LQ and RH, the same PCR product can be used for both tests: LQ detection just needs 4 μL of the amplimer, and RH requires 10 μL. On the other hand, a larger amount of sample is needed for PS test, namely 3 × 75 μL per sample. This can be a limitation for the process, as the samples are supposed to be first analyzed by HC2 (75 μL per sample) and then genotyped by PS. If the protocol needs to be repeated, the remaining material may not be enough.

Regarding working time, LQ system was the one offering the best ratio samples analyzed/time invested; up to 96 samples could be analyzed in a two hours protocol. For the same amount of analyzed samples, LA and RH tests needed to be performed three times, amounting up to six hours. PS required the same time as HC2, but only 28 samples could be analyzed per protocol, so three different plates might be analyzed for obtaining results of 96 samples.

In conclusion, LQ, RH and PS tests showed an overall good concordance for HPVs detection when compared with the FDA approved HR-HC2 system. The three tests displayed type-specific differences with LA genotyping, with PS showing the highest discordance. LQ and RH rendered lower detection rate for multiple infections than LA genotyping. However, our understanding of the clinical significance of multiple HPVs infections is still incomplete and therefore the relevance of the lower ability to detect multiple infections needs to be evaluated.

## Competing interests

JMG, FXB and SdS have received occasional travel fund to attend conferences/symposia/meetings by Qiagen.

## Authors' contributions

JMG conceived the study, participated in the design and coordination, and drafted the manuscript. ST participated in the design of the study and performed the statistical analyses. NB, JMC, MA and ML participated in the design of the study and in samples and data collection. IGB participated in the design and coordination of the study. FXB participated in the design and coordination of the study. SdS conceived the study and participated in the design and coordination. All authors contributed to the final draft of the manuscript. All authors read and approved the final manuscript.
